# Endoscopic Surveillance after (Procto)Colectomy with Gastrointestinal Reconstruction in Patients with Familial Adenomatous Polyposis (FAP)—Principles, Goals and Practical Aspects Based on 12 Years of Observation

**DOI:** 10.3390/life14081000

**Published:** 2024-08-12

**Authors:** Jarosław Cwaliński, Wiktoria Zasada, Hanna Cholerzyńska, Wiktoria Andrzejewska, Hanna Michalak, Tomasz Banasiewicz, Jacek Paszkowski

**Affiliations:** Department of General, Endocrinological Surgery and Gastroenterological Oncology, Poznan University of Medical Sciences, 61-701 Poznań, Poland; 81576@student.ump.edu.pl (W.Z.); hanna.cholerzynska@usk.poznan.pl (H.C.); wiktoria.andrzejewska3@usk.poznan.pl (W.A.); hanna.michalak@usk.poznan.pl (H.M.); tbanasie@ump.edu.pl (T.B.); jpaszkow@ump.edu.pl (J.P.)

**Keywords:** familial adenomatous polyposis, colectomy, endoscopic surveillance, colorectal cancer, follow-up visits

## Abstract

(1) Background: Familial adenomatous polyposis (FAP) is a hereditary condition characterized by the development of numerous adenomas in the large intestine, often necessitating colectomy due to an elevated risk of colorectal cancer. Despite surgical intervention, adenomas frequently recur, underscoring the importance of ongoing surveillance. This study evaluates the outcomes of a 12-year endoscopic follow-up after colectomy and gastrointestinal reconstruction for FAP. (2) Methods: A retrospective analysis was conducted on 41 FAP patients who underwent at least one postoperative endoscopic examination. Assessments of the pouch or rectum were performed every 12–18 months following ileorectal anastomosis and every 18–24 months after ileal pouch–anal anastomosis. Follow-up biopsies were assessed using the adopted Spigelman classification. (3) Results: Postoperative pathology revealed invasive colorectal cancer in three patients. Abdominoperineal resection was performed in two cases due to secondary invasive carcinoma, and one T1 tumor was radically removed with ESD. One patient underwent radical pouch excision following a nodal pelvic recurrence of rectal cancer. Over a 12-year observation period, the mean Spigelman score increased by 2 points, and the proportion of patients with low-grade polypoid lesions decreased. The quantity or size of polyps increased in 24 patients, decreased in 8 patients, and remained stable in 9 patients. In four patients, granular, laterally spreading tumors were discovered in the rectal stump. (4) Conclusions: Regular endoscopic surveillance in FAP patients facilitates early identification of neoplastic and inflammatory changes. The downstaging potential highlights the effectiveness of early interventions. While the Spigelman classification assessed polyps well, it did not predict cancer occurrence. A notable number of patients had invasive cancer at the time of surgery, underscoring the importance of early surgical qualification, which is particularly crucial for identifying upstaging or secondary cancer.

## 1. Introduction

Familial adenomatous polyposis (FAP) represents a distinctive hereditary colorectal disorder, attributed to the presence of an inactivating mutation in the Adenomatous Polyposis Coli (APC) gene, ultimately leading to the development of multiple adenomas within the colon [1]. The diagnosis of this condition commonly manifests at an early age, often due to a positive family history, prompting the necessity for genetic screening in relatives aged 10 to 15, following the guidelines outlined by the American National Comprehensive Cancer Network (NCCN) [2].

The principal challenge in managing patients with confirmed APC mutations is the significant risk of colorectal cancer, which reaches 100% if colectomy is not performed in a timely manner [3,4]. Carcinogenesis typically initiates approximately a decade subsequent to the manifestation of polyp formation [5]. Therefore, vigilant and systematic endoscopic surveillance assumes paramount significance in the ongoing management of this disorder. This surveillance is crucial for monitoring disease progression, discerning neoplastic transformation, and identifying potential inflammatory changes in individuals diagnosed with FAP. Consequently, the recommendation stipulates the adoption of flexible colonoscopy at 12-month intervals for individuals aged 10 to 15 years [6].

Notably, the disconcerting occurrence of sporadic cases of colorectal cancer emerging in patients during their teenage years has been well documented in the scientific literature [6,7]. This underscores the imperative need for heightened awareness and early detection strategies, given the potential for malignancy to manifest at a relatively young age in individuals harboring FAP-related genetic mutations.

In addressing the therapeutic spectrum for FAP patients, a range of surgical interventions presents itself, each with its own distinctive considerations. Options include colectomy with ileorectal anastomosis (IRA), proctocolectomy with end ileostomy, and proctocolectomy with ileal pouch–anal anastomosis (IPAA) [2]. The choice among these interventions is contingent upon factors such as the extent of the disease, patient preferences, and the overarching goal of mitigating the risk of colorectal cancer while preserving optimal gastrointestinal function.

Despite the surgical intervention of colon removal, patients undergoing procedures such as IPAA or IRA may encounter the development of adenomas, and concomitantly, the persistence of severe colorectal polyposis has been identified as a notable risk factor for the emergence of adenomas in the context of IPAA [8,9,10,11]. This underscores the nuanced and dynamic nature of postoperative outcomes in familial adenomatous polyposis (FAP) patients, where the underlying genetic predisposition can influence the ongoing risk of adenomatous growth despite prior colectomy.

However, despite the evident clinical complexities and challenges associated with postoperative scenarios, there remains a conspicuous dearth of comprehensive information regarding the optimal post-surgical management strategies tailored specifically for FAP patients. The multifaceted nature of the factors influencing the postoperative course in these individuals further adds to the existing lacuna in knowledge. This gap in understanding necessitates a concerted effort to unravel the intricacies of post-surgical care for FAP patients, ensuring that clinical interventions are not only effective but also aligned with the unique characteristics of this patient population.

Moreover, an additional impediment to a cohesive understanding of the postoperative landscape in FAP patients lies in the absence of standardized tools for assessing ileal pouch and rectal cuff polyps. This lack of uniformity poses a significant challenge, hindering the ability to conduct objective and comparable evaluations of disease progression across different clinical settings. Consequently, there exists a critical need for the development and implementation of standardized tools that facilitate the systematic assessment of polyp burden in these anatomical regions, thereby enhancing the precision and reliability of postoperative evaluations.

Against this backdrop, the primary objective of the current study is to meticulously scrutinize the 12-year surveillance outcomes of patients who have undergone (procto)colectomy with gastrointestinal tract reconstruction for familial adenomatous polyposis. By doing so, the investigation aspires to shed light on the fundamental principles, overarching goals, and practical considerations intricately associated with post-surgical management in this distinct patient population. Through a comprehensive analysis of the long-term surveillance data, the study endeavors to contribute valuable insights that can inform evidence-based strategies for optimizing the postoperative care trajectory of FAP patients, thereby advancing our understanding and improving clinical outcomes in this challenging medical context.

## 2. Materials and Methods

This retrospective study, conducted within the authors’ Surgery Department from 2010 to 2022, delves into a group of patients who were enrolled for colectomy or proctocolectomy followed by digestive tract reconstruction. This select group underwent a minimum of two endoscopic examinations as integral components of their postoperative follow-up. The driving impetus for surgery in these individuals was rooted in the presence of multiple colorectal adenomas concomitant with a confirmed APC gene mutation, a hallmark association with FAP.

Stratifying the patient cohort based on the extent of rectal adenomas, those without or with only a few were directed towards colectomy with a one-time ileorectal anastomosis (IRA). Conversely, if rectal polyps were also in evidence, the preferred intervention took the form of restorative proctocolectomy with ileal pouch–anal anastomosis (IPAA). In the case of increased intraoperative risk, the strategic creation of a protective stoma was implemented and subsequently closed within the ensuing 3–6 months. The standard pre-surgical protocol involved a sigmoidoscopy, a procedure during which the extent of resection was determined. Patients who underwent only colectomy, i.e., without reconstructive surgery, and those with insufficient follow-up data were excluded from the study.

The subsequent phase of observation involved endoscopic surveillance focusing on the assessment of the pouch or rectum. Following IRA reconstruction, examinations took place at 12–18 month intervals, while the timeline was extended to 18–24 months post-IPAA. In instances where lesions carrying an elevated risk of dysplasia were identified, a bespoke supervisory approach was adopted to ensure individualized care.

The priority of the endoscopic follow-up was to assess the severity (progression/regression) of polyposis and detect the degree of polyp dysplasia, including the risk of malignant transformation. The assessment included: (1) polyp size: the diameter of the base and the length of the polyp, including the stalk for pedunculated polyps; (2) lesion morphology according to the Paris classification, (3) degree of dysplasia obtained through narrow-band imaging, defined by the JNET classification; (4) histological type determined by microscopic examination of biopsy samples or material from endoscopic resection; and (5) severity of polyposis based on the Spiegelman scale, adapted for evaluating pouches or rectums, with mandatory scoring of the number and diameter of polyps. The next two categories of this scale, histology and dysplasia, were assessed through histopathological examination. However, for lesions corresponding to types 1 and 2A on the JNET scale, a biopsy was not mandatory.

Routine endoscopic biopsies were taken in cases of suspected submucosal invasive cancer and high-grade intramucosal neoplasia, corresponding to grades 3 and 2B on the JNET scale, especially if endoscopic resection was deemed challenging or impossible. Each microscopic examination was performed by a pathologist experienced in gastrointestinal diseases. In cases of lesions showing neoplastic infiltration or “questionable” changes, such as unclear resection margins or incomplete/fragmented material, the examination was independently evaluated by two pathologists.

**Figure 1:**
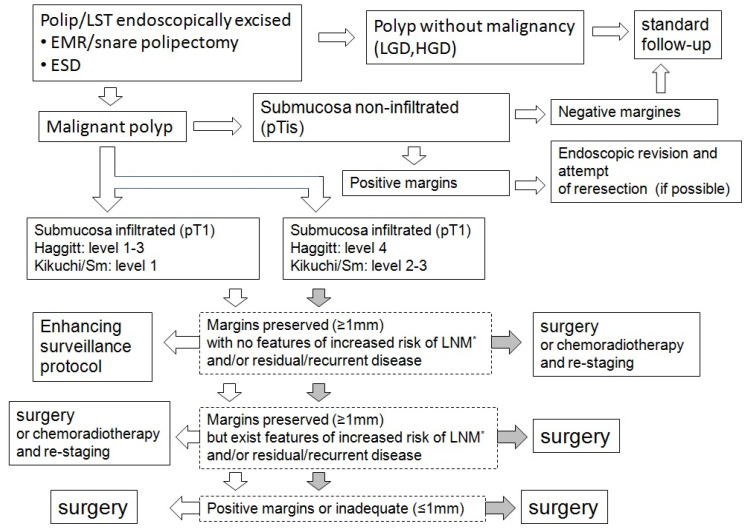
Management of malignant polyps or lateral spreading tumors after endoscopic resection.

Resection was considered radical if negative margins of at least 1 mm were achieved for pT1 lesions and if submucosal penetration did not exceed one-third of its thickness. pT1 lesions excised non-radically or with insufficient margins (<1 mm), as well as poorly differentiated carcinoma and lymphovascular invasion, were referred for radical surgical treatment. Tumor budding, piecemeal resection, and deep SMI, even with negative margins, were also associated with an increased risk of lymph node metastasis (LNM) and/or residual or recurrent disease. However, in these situations, there was a possibility of omitting surgery in favor of chemoradiation (Figure 1). This approach required individualized qualification for oncological treatment, consideration of comorbidities, enhanced surveillance, and patient preference (Table 1).

We adapted the Spiegelman score, which is a tool used to evaluate the severity of duodenal polyposis in patients with familial adenomatous polyposis. It incorporates several criteria, each contributing to the overall score, which guides clinical management and surveillance strategies. The score considers the number of polyps, the size of the largest polyp, histological features, and the degree of dysplasia. Specifically, the number of polyps is categorized as 1–4, 5–20, or more than 20, with increasing scores for higher numbers. The size of the largest polyp is classified into three groups: less than 5 mm, 5–10 mm, and greater than 10 mm, each associated with escalating scores. Histological features are graded from tubular adenoma to tubulovillous and villous adenomas, with higher scores assigned to more advanced histological types. The degree of dysplasia is evaluated as none to mild, moderate, or severe, with higher scores reflecting more severe dysplastic changes.

Within the framework of follow-up therapy, the indications for endoscopic mucosal resection (EMR) or endoscopic submucosal dissection (ESD) hinged on the characteristics of polyps within the pouch, rectum, or rectal stump, specifically those with a diameter exceeding 10 mm. The choice of removal technique was contingent upon the overall dimension and an evaluation based on the Japan NBI Expert Team (JNET) score. Furthermore, resection of adenomas ranging in size from 5 to 10 mm was recommended if the lesions were few in number, a strategic maneuver aiming for the attainment of a completely polyp-free intestine. Lesions penetrating the submucosa and those with a diameter exceeding 20 mm, as well as polyps with 2B and 3 foci according to the JNET classification, were marked with ink for endoscopic follow-up after confirming the radicality of the resection in histopathological examination. This nuanced approach aligns with the imperative of meticulous postoperative care, emphasizing both individualized vigilance and evidence-based interventions in the management of FAP patients undergoing reconstructive surgery.

### 2.1. Statistical Analysis

Statistical analysis was performed using the Statistica 12.0 software (Tibco Inc., Tulsa, OK, USA). Descriptive statistics were applied to measurable variables. Quantitative data were first assessed for normal distribution using the Shapiro–Wilk test. As the data did not exhibit a normal distribution, they were reported as medians. A *p*-value of 0.05 or less was considered statistically significant.

### 2.2. Ethical Statement

The therapeutic approach delineated in our study adhered rigorously to the tenets of medical ethics and the standards of good medical practice. This assertion underscores our commitment to ensuring the welfare, autonomy, and dignity of the individuals participating in the research. The study design itself was predicated on a retrospective analysis, a methodology that obviated the necessity for a distinct and independent consent process from the regional bioethics committee, given the inherent nature of our inquiry. Before the initiation of any medical procedures, patients involved in our study were diligently apprised of the pertinent information, risks, and potential benefits associated with their participation. This comprehensive informed consent process was documented in written form, underscoring the voluntary and autonomous nature of their agreement to partake in the study. The documentation of informed written consent serves not only as an ethical imperative but also as a tangible testament to the transparency and respect accorded to the autonomy of the individuals who contributed to the corpus of our research.

## 3. Results

The study cohort included 41 patients (15 males, 26 females) with a mean age of 31 years (range: 23–38 years). Within this group, 28 cases underwent colectomy with ileorectal anastomosis, while 13 patients opted for proctocolectomy with ileal pouch–anal anastomosis. In 14 instances, a protective ileostomy was deemed necessary. The postoperative pathology unveiled early invasive colorectal cancer in 3 patients, with one of them undergoing simultaneous non-anatomic liver resection due to metastasis.

Throughout the postoperative period, all patients remained under diligent endoscopic surveillance, yet in six cases, the follow-up was deemed incomplete. This incompleteness manifested as the omission of one examination or an extension of the period between examinations from the prescribed 6 to 8 months. The majority of patients received multiple follow-up visits, underscoring the commitment to ongoing monitoring. Specifically, 39 patients had the benefit of two follow-up visits, 29 patients underwent three visits, and 23 patients experienced four or more follow-up visits, highlighting the thoroughness of the surveillance protocol implemented. The interval between surgery and the inaugural follow-up visit was quantified, revealing a median duration of 15 months (ranging from 12 to 24 months). The range encompassed a spectrum from the briefest interval of 3 months to the most protracted interval of 35 months. Furthermore, the median duration of overall follow-up extended to 5.8 years (with a range from 4.0 to 8.1 years).

**Figure 2:**
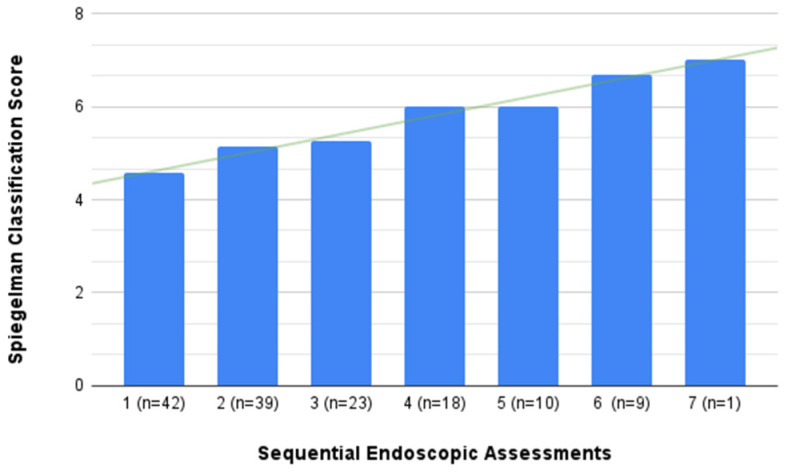
The mean value of the Spiegelmann classification scores was obtained in the following assessments of endoscopic surveillance. In each assessment (n) patients took part. The green line represents the trend.

**Figure 3:**
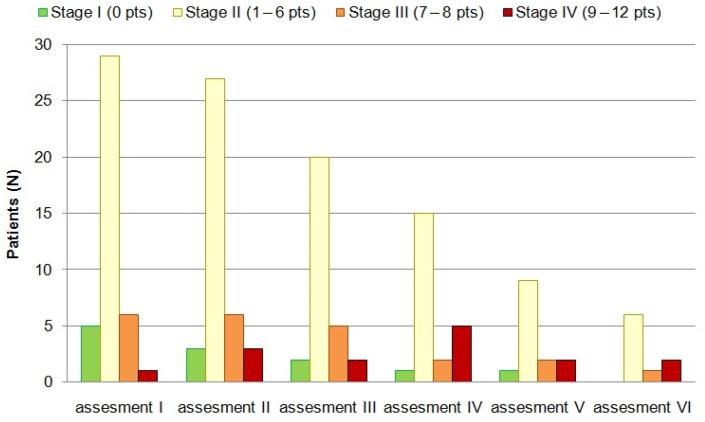
The severity of recurrent adenomatous polyposis of the rectum, pouch, or rectal cuff in the following endoscopic assessments is expressed in Spiegelman classification points and stages.

The Spiegelman score, a critical tool for risk assessment, exhibited an average increase of 2 points after 12 years of meticulous observation. Simultaneously, a discernible trend emerged in the distribution of low-grade polypoid lesions, characterized by a Spiegelman score of 1 to 6, with a consistent decrease observed across consecutive examinations (refer to Figure 2 and Figure 3).

**Figure 4:**
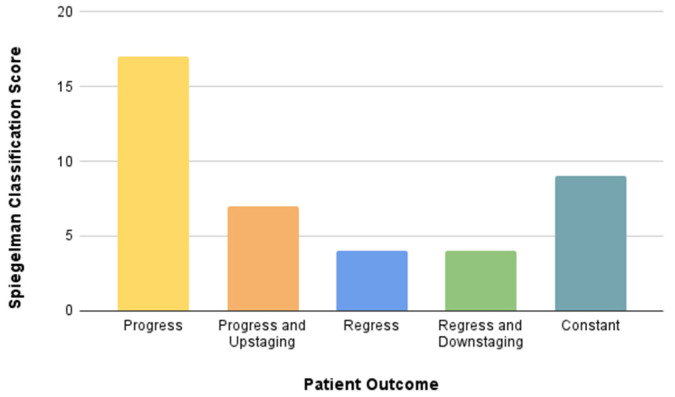
Summary of 12 years of endoscopic surveillance. Patients are divided into five groups depending on the severity of polyps transformation compared to the first examination after GI tract reconstruction. Analysis based on points and degrees of the Spiegelman score.

A consistent and steady increase in either the number or diameter of polyps was documented in 24 patients. However, it is noteworthy that only seven of these individuals necessitated intensified surveillance due to the observed polyp-related changes. On the contrary, a positive trend was discerned in four patients wherein endoscopic resection of polyps led to downstaging, facilitating a return to the baseline observation intervals (Figure 4).

Among the patients who underwent ileal pouch–anal anastomosis (IPAA), a distinctive finding surfaced in the form of a granular laterally spreading tumor (LST-G) identified in the rectal stump. This unique presentation prompted proactive measures, with radical removal achieved through endoscopic mucosal resection (EMR) in two cases. However, in another two patients, the LST lesion proved endoscopically unresectable. Consequently, a biopsy sample was judiciously obtained in successive examinations, and further growth was constrained through the application of argon coagulation.

In response to secondary invasive carcinoma, two patients within the cohort underwent abdominoperineal resection. In another instance, a T1 tumor was successfully and radically removed utilizing endoscopic submucosal dissection (ESD). Notably, a unique case warranted a referral for radical pouch excision. This decision was prompted by the emergence of a nodal pelvic recurrence of rectal cancer four years after the initial colectomy, although notably, no secondary neoplasm was identified in the vicinity of the ileorectal anastomosis (Table 2).

## 4. Discussion

The comprehensive objective of endoscopic surveillance in individuals diagnosed with familial adenomatous polyposis (FAP) and having undergone either ileal pouch–anal anastomosis or ileorectal anastomosis is characterized by a dual focus: the discernment of polyposis recurrence in the pouch or rectum, and, crucially, the prevention of malignancy following polyp dysplasia [12]. The intricate dynamics inherent in this surveillance endeavor come to light when delving into the reported development of pouch adenomas, a spectrum spanning from 25% to 100%, contingent upon variables like the duration of follow-up, lesion progression, and the temporal trajectory since the surgical procedure [11,13,14]. Disparities in results across surveillance centers are attributed to variations in patient characteristics, particularly those entering surgery with more advanced disease, demonstrating a predisposition to pouch adenoma development [11].

An essential facet in the continuum of endoscopic follow-up pertains to the incidence of cancer diagnoses during the surveillance period. Long-term observations highlight a notably elevated risk of secondary rectal cancer in patients with rectum preservation, reaching an incidence of 11% over an average follow-up duration of 15 years [15,16]. In contrast, individuals undergoing ileal pouch–anal anastomosis (IPAA) face a comparatively lower risk, with malignant transformation occurring in up to 2% of cases. Notably, the rectal cuff remains particularly susceptible to polyp formation and subsequent oncogenic transformation [17,18]. The interplay of these factors underscores the need for diligent surveillance strategies, emphasizing the intricate balance between identifying potential malignancies and preventing their onset in this unique patient population.

This multifaceted surveillance landscape necessitates ongoing attention to nuances, as the outcomes are intricately tied to the complex interplay of patient-specific characteristics, surgical interventions, and the underlying genetic predisposition. The reported range of pouch adenoma development and the divergent risks associated with rectal preservation versus IPAA underscore the dynamic nature of postoperative outcomes, necessitating tailored and vigilant surveillance approaches.

Furthermore, the observed differences in cancer incidence rates during the surveillance period accentuate the critical role of surveillance intensity and frequency. The elevated risk of secondary rectal cancer in patients with preserved rectum highlights the imperative for more rigorous surveillance protocols, including frequent and meticulous endoscopic examinations, to detect early signs of malignancy. Conversely, the comparatively lower risk for individuals undergoing IPAA suggests that surveillance efforts can be adjusted to align with the lower likelihood of malignant transformation, albeit without compromising the essential need for ongoing monitoring.

As our understanding of the nuanced dynamics of endoscopic surveillance in FAP patients continues to evolve, it becomes evident that tailored approaches must be developed, taking into account individual patient profiles, surgical histories, and underlying genetic factors. The intricate interplay of these elements demands a holistic and personalized approach to surveillance, ensuring that the frequency, intensity, and methodologies align with the specific risks and characteristics of each patient.

The insights gleaned from a study conducted by Tajika M et al., which mirror the adenoma incidence observed in our study, reveal a noteworthy aspect of the postoperative landscape in familial adenomatous polyposis (FAP) patients. This study reported the development of adenocarcinoma in 4.26% of patients undergoing restorative colectomy [13]. In stark contrast, proctocolectomy with ileal pouch–anal anastomosis (IPAA) demonstrates a remarkable reduction in polyposis recurrence, with malignancy being observed relatively infrequently [11]. This disparity underscores the divergent outcomes associated with distinct surgical interventions, emphasizing the importance of tailoring postoperative management strategies to the specific needs and risks of FAP patients.

Upon closer examination of various surveillance schedules for FAP patients diagnosed with secondary cancer, a temporal pattern in oncogenesis emerges, typically commencing around 6–7 years after colectomy. Intriguingly, the presence of laterally spreading tumor (LST) lesions, while not extensively described in the context of FAP, emerges as a noteworthy phenomenon, despite the implication of APC mutations in its molecular pathogenesis [19,20]. The identification of these lesions, predominantly in the rectal cuff, emphasizes the imperative need for meticulous surveillance strategies to detect and manage potential malignancies in this unique patient population, shedding light on the dynamic and evolving nature of the postoperative risk landscape.

Following general recommendations, individuals who have undergone colectomy with restorative surgery are advised to undergo regular endoscopic surveillance, typically at biennial intervals [21]. However, it is essential to note a divergence in recommendations, with some authors advocating for a more frequent surveillance interval of one year, particularly in patients with an ileal pouch. Aligning with these considerations, our approach involved recommending endoscopic follow-up examinations within the range of 12 to 24 months. The rationale behind this prescribed interval is rooted in the imperative to effectively detect any recurrence or emergence of polyps in the remaining recto-intestinal segments, including the vulnerable rectal cuff, which has been identified as particularly susceptible to polyposis development in individuals with familial adenomatous polyposis.

Despite the meticulously designed surveillance protocols, it is noteworthy that various factors, including the impact of the COVID-19 pandemic, occasionally hindered patients from participating in their scheduled surveillance appointments in a timely manner. This recognition underscores the complexity of implementing standardized surveillance practices in real-world scenarios, where external factors may impact patient adherence and healthcare delivery. Acknowledging that the decision to attend follow-up examinations ultimately rests with the patient is of paramount importance, highlighting the delicate balance between medical guidance and patient autonomy. Despite the critical nature of regular examinations, patients maintain autonomy in determining whether or not to schedule and attend these follow-up appointments [22].

The standardization of endoscopic examination results constitutes a pivotal aspect in the comprehensive management of familial adenomatous polyposis (FAP), relying heavily on the judicious assessment of polyps according to classifications specifically designed for endoscopy [23,24]. While established scales such as the Paris and Japan NBI Expert Team scales prove valuable for characterizing individual lesions, they face some limitations when applied to the description of the multitude of polyps typical of FAP. From this nuanced perspective, the Spiegelman classification emerges as a more fitting and practical option for FAP patients, offering a comprehensive characterization of the entire polyp population within the examined part of the digestive tract [25]. Originally designated for the duodenum, the Spiegelman score demonstrates its versatility by being effectively applied in endoscopy of the intestinal pouch and rectum, presenting itself as a viable alternative for routine follow-up examinations.

The Spiegelman classification incorporates four fundamental criteria—number of polyps, size, morphology, and degree of dysplasia—all intricately linked to neoplasia. Consequently, the Spiegelman score serves as a credible tool for risk assessment and guiding treatment decisions in the context of FAP. Its advantages extend beyond a mere diagnostic role, including the facilitation of result comparison across consecutive endoscopies. This functionality proves invaluable in tracking the progression or regression of lesions over time, offering clinicians a dynamic understanding of the patient’s evolving condition [26,27]. Additionally, the Spiegelman score plays a crucial role in confirming downstaging rates following polyp removal, contributing to the ongoing assessment of the efficacy of interventions [27].

However, our study illuminates several limitations associated with the application of the Spiegelman scale in the context of FAP. Notably, a high score on this scale does not consistently correlate with a high risk of secondary cancer formation [26,28]. This observation seems to be linked to the potential underestimation of the degree of dysplasia in the final score. Theoretical scenarios, such as two patients—one with numerous small polyps exhibiting small dysplasia and the other with only one polyp but harboring large dysplasia—may result in the same score on the Spiegelman scale, despite the higher risk of cancer development in the latter case [28]. Instances in our study where patients who developed malignancy achieved only 8 points on the Spiegelman scale underscore the imperative for nuanced interpretation and the consideration of additional factors in the complex landscape of risk assessment.

Another benefit of endoscopic surveillance, apart from detecting the severity of polyposis, is the removal of polyps presenting with high-grade dysplasia (HGD) or invasive adenocarcinoma. Adenomas characterized only by intramucosal malignancy, i.e., not infiltrating the submucosal layer, are defined as pTis according to the TNM staging system. In these situations, standard polypectomy usually provides negative margins and can be considered a curative procedure. Conversely, malignant polyps (MP), referred to as pT1, invade the muscularis mucosae but do not penetrate beyond the muscularis propria. The main indicator of radical excision is the depth of submucosal infiltration, which corresponds to the degree of risk for lymph node metastasis (LNM) and/or residual or recurrent disease [29,30].

Therefore, in addition to TNM staging and achieving negative margins (not less than 1 mm), the polyp should be classified according to the Haggitt/Kikuchi scale. Additional risk factors should also be determined, such as poorly differentiated carcinoma, lymphovascular invasion, tumor budding, piecemeal (incomplete) excision, and deep submucosal invasion (SMI). Malignant polyps with neoplastic invasion between levels 1 and 3 according to Haggitt or Sm1 according to Kikuchi, removed with a margin of ≥1 mm, can be considered fully radical. However, the patient is subject to enhanced follow-up through endoscopy and imaging studies in the following months. In other situations, surgical resection (transabdominal or transanal) is recommended, although some centers allow radiochemotherapy as a strictly individualized treatment [29,31,32].

The intricate challenge associated with describing and classifying laterally spreading tumor-type lesions, particularly their dynamic growth or regression observed in subsequent endoscopies conducted as part of surveillance, stands out as a noteworthy concern in the management of familial adenomatous polyposis (FAP). The expansive nature and heterogeneous structure of these lesions, whether granular or non-granular, present a unique set of complexities, and the description provided by the Spiegelmann scale is often incomplete. This limitation has the potential to lead to an underestimation of the predicted risk, highlighting the need for a more nuanced and comprehensive approach to capturing the intricacies of these lesions within the diagnostic framework [33].

In the realm of therapeutic endoscopy, a pivotal role is assumed in the surveillance of FAP patients, contributing not only to disease stabilization but also to the regression of lesions in a subset of cases. While the downstaging achieved on the Spiegelman scale may not uniformly correlate with a reduced risk of malignancy, the removal of polyps exceeding 10 mm and villous polyps imparts considerable benefits [34]. However, it is important to acknowledge a limitation inherent in this classification, specifically its uniform allocation of scores to various components. For instance, it assigns 3 points to both scenarios of numerous polyps and a solitary polyp exhibiting high dysplasia. From a cancer risk perspective, these scenarios are not equivalent, as illustrated by cases where patients with cancer scored only 6 points on the Spiegelman classification [28]. This discrepancy emphasizes the complexity of risk assessment in the context of FAP and the need for a more nuanced scoring system that accounts for the varying clinical implications of different scenarios.

The radical excision of laterally spreading tumor (LST) lesions often proves impractical, and partial excision tends to be ineffective, particularly as resection usually targets only the peripheral part of the lesion, where the degree of dysplasia is lower than in the center [34,35]. An alternative approach involves obtaining multiple biopsies from the lesion and shortening the time between examinations to 3–6 months based on the pathology result. In cases of advanced LST changes and persistent local recurrence of polyps, argon coagulation serves as a last-resort remedy. While this technique yields a satisfactory local effect leading to stromal fibrosis, a disadvantage lies in the lack of complete material for pathology examination, potentially increasing the risk of unnoticed malignancy [36].

The intricacies of managing laterally spreading tumor-type lesions underscore the evolving nature of therapeutic strategies in FAP patients. The limitations associated with existing classifications necessitate ongoing research and the development of more refined diagnostic and therapeutic modalities to address the diverse challenges posed by these lesions. As our understanding deepens, a comprehensive and tailored approach to managing these lesions will contribute to enhanced outcomes and a more effective surveillance framework in the complex landscape of familial adenomatous polyposis.

## 5. Limitations

Notwithstanding the valuable insights gleaned from our study, it is essential to acknowledge several inherent limitations that may impact the robustness of our findings. Firstly, our study relies partially on retrospective data, which introduces the possibility of inherent biases and may limit the coherent interpretation of the results. The reassessment of data using the latest endoscopic scales, while enhancing the applicability of contemporary standards, also introduces potential challenges associated with historical data accuracy and consistency. Secondly, the relatively modest sample size utilized in our study imposes constraints on the generalizability of our findings. The inherent variability within a smaller cohort may limit the extent to which our results can be extrapolated to the broader population of individuals with familial adenomatous polyposis. Consequently, caution should be exercised in making sweeping generalizations about the broader FAP patient population based solely on our study’s outcomes. Additionally, the single-clinic setting employed in our study may further impact the external validity of our results. The unique patient demographics, treatment protocols, and clinical practices within a single clinic may not be fully representative of the diverse contexts in which FAP patients receive care. Consequently, the generalizability of our findings to other clinical settings and populations should be approached with circumspection. In light of these acknowledged limitations, it is imperative for future research endeavors to incorporate larger and more diverse cohorts, employ prospective study designs, and consider multi-center collaborations. Such approaches would contribute to a more comprehensive understanding of the nuanced dynamics associated with FAP management, thereby fostering advancements in the field and refining the evidence base for optimal patient care.

## 6. Conclusions

In conclusion, our comprehensive investigation into postoperative management and endoscopic surveillance for familial adenomatous polyposis underscores the crucial significance of regular endoscopic surveillance in individuals who have undergone colectomy or proctocolectomy. This surveillance plays a pivotal role in facilitating the early detection of malignancy and inflammatory changes, thereby contributing to improved patient outcomes. Notably, a subset of patients within our study presented with invasive cancer at the time of surgery, emphasizing the imperative for earlier surgical qualification and intervention.

However, our study also reveals the existing gaps in our understanding of FAP management, urging the need for further research with larger sample sizes and standardized assessment tools. Enhancing our knowledge in these areas is vital for refining risk assessment tools and optimizing the care trajectory for this high-risk population. Emphasis on patient education and strict adherence to surveillance guidelines emerge as potential avenues to alleviate the cancer burden in individuals with FAP. The findings from our study serve as a foundation for ongoing efforts to advance the field, ultimately aiming to enhance the overall management and outcomes for patients with familial adenomatous polyposis.

## Figures and Tables

**Table 1:** Enhanced surveillance protocol after endoscopic removal of an adenoma penetrating the submucosa (pT1, Sm1) with margins preserved (≥1 mm) and no additional features of increased-risk LNM and/or residual/recurrent disease.

Type of Examination	Time Interval
Flex endoscopy	3-3-6 months for the first year, then every 1 year
CEA, AFP markers	3-3-6 months for the first year, then every 1 year
Pelvic MRI with assessment of pouch or rectum	Every 6 months for the first year, then every 1 year
Chest and abdominal CT	Every 6 months for the first year, then every 1 year

**Table 2:** Summary of endoscopic surveillance for patients with secondary colorectal cancer.

Sex/Age (at the End of Follow-Up)	Type of Procedure (IRA or IPAA)	Time of Follow-Up (Years)	SC for 1. Exam	SC for 2. Exam	SC for 3. Exam	SC for 4. Exam	SC for 5. Exam
M/45	IRA	5	8	5	5	8 *	-
M/55	IPAA	10	10	12	12	11	12
W/54	IRA	5	5	6	4	4 **	-
W/27	IRA	6	7	7	6	9	-

* high-grade dysplasia with invasive cancer in a tubular polyp. ** nodal pelvic recurrence without confirmed secondary neoplasm in the ileorectal anastomosis.

## Data Availability

The data presented in this study are available on request from the corresponding author.
